# Crosstalk between transforming growth factor β-2 and Autotaxin in trabecular meshwork and different subtypes of glaucoma

**DOI:** 10.1186/s12929-021-00745-3

**Published:** 2021-06-17

**Authors:** Nozomi Igarashi, Megumi Honjo, Reiko Yamagishi, Makoto Kurano, Yutaka Yatomi, Koji Igarashi, Toshikatsu Kaburaki, Makoto Aihara

**Affiliations:** 1 Department of Ophthalmology, Graduate School of Medicine, The University of Tokyo, Tokyo, Japan; 2 Department of Clinical Laboratory Medicine, Graduate School of Medicine, The University of Tokyo, 7-3-1 Hongo Bunkyo-ku, Tokyo, 113-8655 Japan; 3 CREST, Japan Science and Technology Corporation (JST), Saitama, Japan; 4 Department of Clinical Laboratory, The University of Tokyo Hospital, Tokyo, Japan; 5 Bioscience Division, Reagent Development Department, AIA Research Group, TOSOH Corporation, Kanagawa, Japan; 6 Department of Ophthalmology, Jichi Medical University Saitama Medical Center, Saitama, Japan

**Keywords:** Transforming Growth factor β, Autotaxin, Glaucoma, Human trabecular meshwork

## Abstract

**Background:**

Elevated transforming growth factor (TGF)-β2 in aqueous humor (AH) has been suggested to contribute to trabecular meshwork (TM) fibrosis and intraocular pressure (IOP) regulation in primary open-angle glaucoma (POAG), but TGF-β2 is downregulated in secondary open-angle glaucoma (SOAG). Because autotaxin (ATX) is upregulated in SOAG, we investigated the relationships and trans-signaling interactions of these mediators.

**Methods:**

The level of ATX in AH was determined using a two-site immunoenzymetric assay, and TGF-β levels were measured using the Bio-Plex Pro TGF-β Assay. RNA scope was used to assess the expression of ATX and TGF-β2 in human’s eye specimen. And in vitro studies were performed using hTM cells to explore if trans-signaling of TGF-β2 regulates ATX expressions.

**Results:**

TGF-β2/ATX ratio was significantly high in AH of control or POAG compared with SOAG, and negatively correlated with IOP. RNA scope revelated positive expressions of both TGF-β2 and ATX in ciliary body (CB) and TM in control, but ATX expressions was significantly enhanced in SOAG. In hTM cells, ATX expressions were regulated by TGF-β2 with concentration-dependent manner. In counter, ATX also induced TGF-β1, TGF-β2 and TGFBI upregulations and activation of the Smad-sensitive promoter, as well as upregulation of fibrotic markers, and these upregulation was significantly suppressed by both TGF-β and ATX inhibition.

**Conclusions:**

Trans-signaling of TGF-β2 regulates ATX expressions and thereby induced upregulations of TGF-βs or fibrosis of hTM. TGF-β2 trans-signaling potently regulate ATX transcription and signaling in hTM cells, which may reflect different profile of these mediators in glaucoma subtypes.

*Trial Registration* This prospective observational study was approved by the Institutional Review Board of the University of Tokyo and was registered with the University Hospital Medical Information Network Clinical Trials Registry of Japan (ID: UMIN000027137). All study procedures conformed to the Declaration of Helsinki. Written informed consent was obtained from each patient.

**Supplementary Information:**

The online version contains supplementary material available at 10.1186/s12929-021-00745-3.

## Background

Glaucoma is the second leading cause of blindness worldwide, characterized by aberrant increases in intraocular pressure (IOP) that can damage the optic nerve [1–3]. Reduction of IOP is the only effective therapy to prevent visual impairment and blindness in both hypertensive and normotensive individuals. IOP is determined by the balance between aqueous humor (AH) production and outflow, and elevated IOP is caused mainly by increased resistance to aqueous outflow. Notably, transforming growth factor (TGF)-β2 is elevated in eyes with primary open-angle glaucoma (POAG), the most common type of glaucoma. In these eyes, TGF-β2 increases aqueous resistance by inducing upregulation of the extracellular matrix (ECM) in the trabecular meshwork (TM). Thus, TGF-β2 is presumed to play an important role in POAG pathogenesis by regulating the fibrotic response of TM cells and expression of ECM proteins through the canonical Smad pathway and other noncanonical signaling pathways [4–7]. TGF-β2 is known to increase expression levels of α-smooth muscle actin (SMA), fibronectin, COL1A1, and other fibrotic markers in TM cells, suggesting that fibrotic regulation by TGF-β2 in the TM may be related to POAG pathogenesis [8, 9].

However, past reports have demonstrated that TGF-β2 is upregulated in patients with POAG, but downregulated in patients with secondary open-angle glaucoma (SOAG) [4, 10]. Thus, we sought other mediators involved in glaucoma pathogenesis, and found that aqueous autotaxin (ATX) and lysophosphatidic acid (LPA) concentrations were significantly correlated with IOP and higher in patients with SOAG [11, 12]. LPA is produced predominantly by the generating enzyme ATX and has been recognized as a major bioactive lipid mediator influencing fibrosis, which enhances outflow resistance in anterior segment perfusion organ culture models [13]. ATX is a glycoprotein involved in various physiological processes, such as fibrosis and cancer survival [14–17]. We also reported that ATX was significantly upregulated during cytomegalovirus (CMV) infection in TM cells (an in vitro model that mimics SOAG), while the expression of TGF-β2 was downregulated upon CMV infection: of note, in CMV DNA-positive AH from patients with Posner-Schlossmann syndrome (an SOAG subtype frequently accompanied by refractory glaucoma with prolonged extensive IOP elevation), the levels of ATX and TGF-β1 in AH were upregulated, while the level of TGF-β2 was downregulated. Moreover, levels of ATX and TGF-β1 in AH were significantly correlated [18]. Hence, we speculated that crosstalk might exist between ATX and other isoforms of TGF-β. To the best of our knowledge, no clinical or in vitro studies have investigated the relationship and crosstalk between ATX and TGF-β signaling. In this study, we evaluated the association between aqueous levels of ATX and TGF-βs among glaucoma subtypes and characterized the interactions of ATX and TGF-β2 signaling in TM cells in vitro.

## Methods

### Patients and aqueous humor collection

AH samples were obtained from patients with cataract or glaucoma who were aged ≥ 20 years and who had undergone cataract surgery or glaucoma surgery from March 2014 to July 2018 at the University of Tokyo Hospital. This prospective observational study was approved by the Institutional Review Board of the University of Tokyo and was registered with the University Hospital Medical Information Network Clinical Trials Registry of Japan (ID: UMIN000027137). All study procedures conformed to the Declaration of Helsinki. Written informed consent was obtained from each patient. Table 1 lists patient characteristics. All patients with open-angle glaucoma were classified into three groups, as previously described [12]. Briefly, patients with substantially elevated IOP and normal IOP without any cause who had a glaucomatous visual field or optic disc, as well as a normal angle with gonioscopy were diagnosed with POAG; patients with inflammatory diseases, chronic postoperative IOP elevation or inflammatory angle findings were diagnosed with SOAG; and patients with pseudoexfoliation materials were diagnosed with XFG. Individuals with cardiovascular disease were excluded from this study. Exclusion criteria included other types of glaucoma (e.g., primary angle-closure glaucoma or congenital/developmental glaucoma) and a previous history of intraocular surgery other than small incision cataract surgery without complications. IOP was determined using a Goldmann tonometer, and the maximum preoperative IOP was evaluated within 3 months prior to surgery. When both eyes in a patient met the inclusion criteria, only the eye treated first was included in the analyses. For all patients, the anterior eye segment including gonioscopy and IOP measurement and optic disc were examined by glaucoma specialists using a slit lamp biomicroscope and dilated fundoscopy to diagnose glaucoma.

**Table 1:** Demographic characteristics of the study population

Variables	Control	POAG	SOAG	XFG	*P-*value
Patients (n)	20	20	19	19	
Number of eyes (n)	20	20	19	19	
Gender (male: female)	5:15	13:7	13:6	12:7	0.0204*
Age (years)					
Mean ± SD	73.5 ± 7.1	71.2 ± 10.2	61.2 ± 14.8	75.6 ± 12.8	< .005^†, ††††^
[range]	58–87	52–87	39–87	39–93	
IOP (mmHg)					
Mean ± SD	13.6 ± 1.7	15.7 ± 4.0	22.4 ± 11.9	29.7 ± 9.3^†^ < .001,	< .0001**^†, †††^
[range]	10–16	8–25	7–52	13–48	

*POAG* primary open angle glaucoma, *SOAG* secondary open angle glaucoma, *XFG* exfoliation glaucoma, *IOP* intraocular pressure, *ATX* autotaxin, *TGFβ* transforming growth factor beta

^*^Fisher's exact test; **Kruskall-Wallis test

^†^Statistically significant difference between control and SOAG (Steel–Dwass test)

^††^Statistically significant difference between control and XFG (Steel–Dwass test)

^†††^Statistically significant difference between POAG and XFG (Steel–Dwass test)

^††††^Statistically significant difference between SOAG and XFG (Steel–Dwass test)

AH samples were collected as described previously [11, 12, 18]. Briefly, preoperative AH was obtained at the start of the surgery before any incisional procedures, with patients under topical anesthesia. Approximately 70–100 μL AH was obtained using a 30-gauge syringe, collected in a PROTEOSAVE SS 1.5-mL Slimtube (Sumitomo Bakelite, Tokyo, Japan), registered, and stored at − 80 °C until processing.

### Measurement of ATX, ATX isoforms, and TGF-β1, 2 and 3 in AH or cell culture medium

The level of ATX in AH was determined using a two-site immunoenzymetric assay with an ATX assay reagent by means of a Tosoh AIA system (Tosoh, Tokyo, Japan), as described previously [11, 12, 18]. TGF-β levels in AH were measured using the Bio-Plex Pro TGF-β Assay (Bio-Rad, Hercules, CA, USA), in accordance with the manufacturer’s protocol. LysoPLD activity in the culture medium was determined as previously described [11, 12, 18].

### RNAscope analysis

Whole-eye specimens were obtained from patients who underwent autopsy at Singapore Eye Institute. The specimens were immediately fixed in 10% buffered neutral formalin and embedded in OCT compound (Tissue-Tek OCT Compound, Sakura Finetek Japan Co., Ltd., Tokyo, Japan). The eyes were obtained and managed in compliance with the tenets of the Declaration of Helsinki. Ten-micrometer-thick cryosections were cut at − 20 °C and stored until processing.

RNAscope analysis was performed using RNAscope® Multiplex Fluorescent Assay v2 (Advanced Cell Diagnostics USA, Newark, CA, USA), in accordance with the manufacturer’s protocol. Probes against ATX and TGF-β2 were used.

### hTM cell culture

Primary hTM cells were isolated from human donor eyes and characterized as described previously, in accordance with the method of Keller et al. [19]. They were cultured in Dulbecco’s modified Eagle’s medium containing 10% fetal bovine serum and antibiotic–antimycotic solution (100 ×) (Sigma-Aldrich, St. Louis, MO, USA) at 37 °C in 5% CO_2_. Cells from passages 3–6 were used in the experiments. Cells were treated with TGF-β2, ATX, or LPA with or without the corresponding inhibitors (SB431542, HA130, or Ki16425), or with Smad3 inhibitor (SIS3).

### Virus infection

Cell-free CMV medium was prepared as previously described. [18] The harvested medium was used after one freeze/thaw cycle. After they had reached confluence, hTM cells were incubated with cell-free CMV medium for 2 h at 37 °C in 5% CO_2_ at a multiplicity of infection of 1. After 2 h, the medium was removed, the infected cells were washed twice with phosphate-buffered saline, and fresh growth medium was added containing TGF-β2 or SB431542.

### RT-qPCR

Cells were lysed using ISOGEN (NIPPON GENE, Tokyo, Japan), and mRNA was isolated using chloroform and isopropyl alcohol. The mRNA was treated with a PrimeScript RT Reagent Kit (Takara Bio, Shiga, Japan) to synthesize cDNA. mRNA levels were quantified as previously described [11, 18]. For qPCR, primer sequences were taken from previously published reports, and the primers were purchased from Hokkaido System Science (Hokkaido, Japan).

The sequences of the PCR primers were: GAPDH, forward, 5′-GAGTCAACGGATTTGGTCGT-3′ and reverse, 5′-TTGATTTTGGAGGGATCTCG-3′; ATX, forward, 5′-ACAACGAGGAGAGCTGCAAT-3′ and reverse 5′-AGAAGTCCAGGCTGGTGAGA-3′; TGF-β1, forward, 5′- CCCAGCATCTGCAAAGCTC-3′ and reverse 5′- GTCAATGTACAGCTGCCGCA; TGF-β2, forward, 5′- TGCCGCCCTTCTTCCCCTC-3′ and reverse 5′- GGAGCACAAGCTGCCCACTGA-3′; TGF-β3, forward, 5′- GGTTTTCCGCTTCAATGTGT, and reverse 5′-TATAGCGCTGTTTGGCAATG; TGF-β-induced protein, forward 5′- GTCCACAGCCATTGACCTTT-3′ and reverse 5′- GAGTTTCCAGGGTCTGTCCA-3′; fibronectin, forward, 5′-AAACCAATTCTTGGAGCAGG-3′ and reverse, 5′-CCATAAAGGGCAACCAAGAG-3′; COL1A1: forward, 5′-CAGCCGCTTCACCTACAGC-3′ and reverse, 5′-TTTTGTATTCAATCACTGTCTTGCC-3′; α-SMA, forward, 5′-CCGACCGAATGCAGAAGGA-3′ and reverse, 5′-ACAGAGTATTTGCGCTCCGAA-3; and CTGF, forward, CTCCTGCAGGCTAGAGAAGC-3′ and reverse, 5′- GATGCACTTTTTGCCCTTCTT-3′. The data were normalized relative to GAPDH.

### Immunocytochemistry

Immunocytochemistry was performed as previously described [11, 18]. Cells were grown in chamber slides. hTM cells were fixed in ice-cold 4% paraformaldehyde at 24 h after application of TGF-β2 or SB431542 (Fujifilm, Osaka, JAPAN). The primary antibody was anti-ENPP2 antibody [5H3] (1:1,000; Abcam, Cambridge, MA, USA). The corresponding Alexa Fluor 488 secondary antibody (1:1,000) was purchased from Thermo Fisher Scientific (Waltham, MA, USA). Images were acquired using a BX51 fluorescence microscope (Olympus, Tokyo, Japan).

### Measurement of STAT3 (pY705) and total STAT3 in the cell lysate

Levels of phosphorylated STAT3 and total STAT3 in the cell lysate were measured using a STAT3 (pY705) + total STAT3 enzyme-linked immunosorbent analysis kit (Abcam), in accordance with the manufacturer’s protocol.

### WB analysis

At 1 day post-infection, cells were collected in radioimmunoprecipitation assay buffer (Thermo Fisher Scientific) containing protease inhibitors (Roche Diagnostics, Basel, Switzerland), sonicated, and centrifuged. Protein concentration measurement and sodium dodecyl sulfate polyacrylamide electrophoresis were performed as previously described [11, 18]. Protein bands were transferred to polyvinylidene difluoride membranes (Bio-Rad) and the membranes were immersed in Tris-buffered saline with Tween 20 containing primary antibody. After the membranes had been washed, they were immersed in Tris-buffered saline with Tween 20 containing secondary antibody and reacted with enhanced chemiluminescence (ECL) substrate (Thermo Fisher Scientific). Protein bands were detected using an ImageQuant LAS 4000 mini (GE Healthcare, Chicago, IL, USA). The primary antibodies were anti-TGF-β2 (anti-ENPP2 (1:1000; Abcam), anti-β-tubulin (1:1000; Wako Pure Chemical Industries, Ltd., Osaka, Japan), anti-phospho-STAT3 (Tyr 705) (1:1000; Cell Signaling Technology, Inc., Danvers, MA, USA), anti-STAT3 (79D7) (1:1000; Cell Signaling Technology, Inc.), anti-phospho-SAPK (stress-activated protein kinase)/JNK (Jun amino terminal kinase) (Thy 183/Tyr185) (1:1000; Cell Signaling Technology, Inc.), and anti-SAPK/JNK (1:1000; Cell Signaling Technology, Inc.). A horseradish peroxidase-conjugated secondary antibody (1:2000; Thermo Fisher Scientific) was used to detect the bands. All bands were quantified using ImageJ software (ver. 1.49, NIH, Bethesda, MD, USA).

### Luciferase assay

The luciferase assay was performed as previously described [20, 21]. Briefly, transcriptional activity was assessed following transfection of a luciferase reporter gene fused with CAGA-12. A plasmid containing Renilla luciferase (pRLTK; Promega, Madison, WI) was co-transfected as an internal control. hTM cells were transfected at 80% confluence using Gene Juice® transfection reagent (Merck Millipore, Billerica, MA, USA), in accordance with the manufacturer’s protocol. hTM cells were grown in 6-well plates for 1 day before transfection. At 24 h after transfection, the medium was changed to serum-free Dulbecco’s modified Eagle’s medium. Cells were then stimulated for 24 h with TGF-β, ATX, or LPA, with or without the corresponding inhibitors.

### Statistical analysis

Data were statistically analyzed using the EZR program (Saitama Medical Center, Hidaka, Japan) [22]. The results were expressed as means ± standard deviations (SDs). The *t*-test and chi-squared test or Fisher’s exact test were used for comparisons of two variables. The Steel–Dwass test was used for comparisons of multiple variables. Differences among groups were analyzed by one-way analysis of variance and Tukey’s post hoc test. A value of *P* < 0.05 was considered statistically significant.

## Results

### Levels of TGF-βs and ATX in AH among glaucoma subtypes

**Fig. 1:**
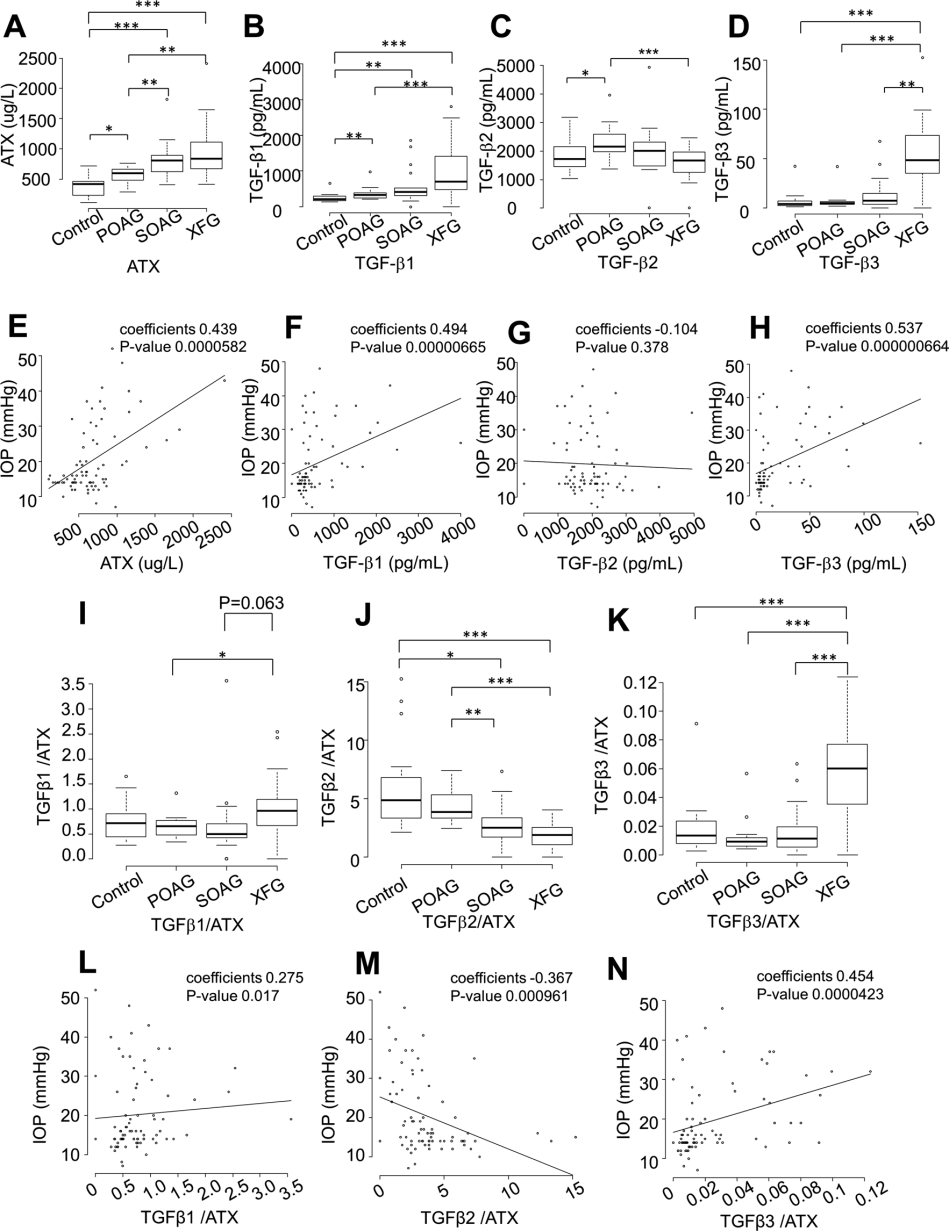
Relationships between glaucoma subtypes and levels of aqueous ATX (**A**), TGF-β1 (B), TGF-β2 (**C**), and TGF-β3 (**D**) and correlations between IOP and levels of aqueous ATX (**E**), TGF-β1 (**F**), TGF-β2 (**G**), and TGF-β3 (**H**). **A** The level of ATX measured by immunoenzymetric assay was significantly lower in the control group than in any glaucoma group (P < 0.05 for POAG, P < 0.001 for SOAG or XFG), and differed significantly between the POAG group and SOAG or XFG groups (P < 0.01). **B** The level of TGF-β1 was significantly lower in the control group than in any glaucoma group (P < 0.01 for POAG and SOAG, P < 0.001 for XFG), while it was higher in the XFG group than in POAG or SOAG groups. **C** The level of TGF-β2 exhibited an inverse tendency, especially between POAG and XFG groups (P < 0.05 between control and POAG, P < 0.001 between POAG and XFG). **D** The level of TGF-β3 was significantly higher in the XFG group than in any other groups (P < 0.01 for SOAG, P < 0.001 for control or POAG). *P < 0.05, **P < 0.01, ***P < 0.01. Levels of ATX (**E**; Spearman’s rank correlation coefficient = 0.439, P = 0.0000582), TGF-β1 (F; Spearman’s rank correlation coefficient = 0.494, P = 0.00000665), and TGF-β3 (**H**; Spearman’s rank correlation coefficient = 0.537, P = 0.000000664) were positively correlated with IOP, whereas the level of TGF-β2 was not (G; Spearman’s rank correlation coefficient = -−0.104, P = 0.378). Relationships between glaucoma subtypes and ratios of aqueous TGF-β1/ATX (**I**), TGF-β2/ATX (**J**), and TGF-β3/ATX (**K**) and correlations between IOP and ratios of TGF-β1/ATX (L), TGF-β2/ATX (M), and TGF-β3/ATX (N). **I** The TGF-β1/ATX ratio was significantly higher in the XFG group than in the POAG group (P < 0.05). **J** The TGF-β2/ATX ratio was significantly higher in the control and POAG groups than in the SOAG or XFG groups (P < 0.05 between control and SOAG, P < 0.01 between POAG and SOAG, P < 0.001 between XFG and control or POAG). **K** The TGF-β3/ATX ratio was significantly higher in the XFG group than in any other group. *P < 0.05, **P < 0.01, ***P < 0.01. **L**–**N** Correlations between IOP and ratios of TGF-β1/ATX, TGF-β2/ATX, and TGF-β3/ATX. Ratios of TGF-β1/ATX (**L**; Spearman’s rank correlation coefficient = 0.275, P = 0.017) and TGF-β3/ATX (**N**; Spearman’s rank correlation coefficient = 0.454, P = 0.0000423) were positively correlated with IOP, whereas the ratio of TGF-β2/ATX was negatively correlated with IOP (**M**; Spearman’s rank correlation coefficient = −0.367, P = 0.000961)

This study included 78 eyes of 78 patients: 20 eyes without any ocular complications (controls), 20 POAG eyes, 19 SOAG eyes, and 19 exfoliative glaucoma (XFG) eyes. Table 1 lists demographic characteristics of the study population. Patients were significantly younger in the SOAG group than in the control or XFG groups (Table 1, P < 0.05). The preoperative IOP was significantly higher in SOAG and XFG groups than in the control group (Table 1, P < 0.01 for SOAG, P < 0.001 for XFG). IOP also differed significantly between POAG and XFG groups (Table 1, P < 0.001). The aqueous level of ATX was significantly lower in the control group than in any glaucoma group (Fig. 1A, P < 0.05 for POAG, P < 0.001 for both SOAG and XFG). The aqueous level of ATX also differed significantly between the POAG group and the SOAG or XFG groups (Fig. 1A, P < 0.01), which was consistent with our previous findings. [12] The aqueous level of TGF-β1 was significantly lower in the control group than in any glaucoma group (Fig. 1B, P < 0.01 for both POAG and SOAG, P < 0.001 for XFG). The XFG group had significantly higher levels of TGF-β1 than the other glaucoma groups. The aqueous level of TGF-β3 was significantly higher in the XFG group than in any other group (Fig. 1D, P < 0.01 for SOAG, P < 0.001 for both control and POAG). The aqueous levels of TGF-βs and ATX differed significantly among glaucoma subtypes. Similar tendencies were observed concerning the levels of TGF-β1, 3, and ATX, while the level of TGF-β2 had an inverse tendency, especially between POAG and XFG groups (Fig. 1C).

### Correlations between IOP and levels of TGF-βs and ATX

Next, we evaluated the correlations of IOP with aqueous levels of ATX, TGF-β1, TGF-β2, and TGF-β3. Figure 1E–H presents the correlations of IOP with aqueous levels of ATX and TGF-β1–3. In the overall cohort, levels of ATX (Fig. 1E, P = 0.0000582), TGF-β1 (Fig. 1F, P = 0.00000665), and TGF-β3 (Fig. 1H, P = 0.000000664) were significantly positively correlated with IOP, while the level of TGF-β2 (Fig. 1G, P = 0.378) was not correlated with IOP. Analysis limited to patients with POAG did not reveal a significant positive correlation between the level of TGF-β2 and IOP, although a positive slope was observed (Additional file 1: Figure S1), which was consistent with previous findings. [23].

### Diagnostic values of TGF-β/ATX ratios among glaucoma subtypes and correlations with IOP

Because aqueous levels of TGF-βs and ATX differed among groups (Fig. 1), especially between POAG and XFG, we next focused on the TGF-β/ATX ratios among glaucoma subtypes and evaluated their diagnostic values. As shown in Fig. 1I, the TGF-β1/ATX ratio was significantly higher in the XFG group than in the POAG group. The TGF-β2/ATX ratio was significantly higher in both the control and POAG groups than in the SOAG or XFG groups (Fig. 1J). The TGF-β3/ATX ratio was significantly higher in the XFG group than in any other groups (Fig. 1K). IOP was significantly positively correlated with both TGF-β1/ATX and TGF-β3/ATX ratios (Fig. 1L [TGF-β1/ATX], P-value = 0.017; Fig. 1N [TGF-β3/ATX], P-value = 0.0000423). The TGF-β2/ATX ratio was significantly negatively correlated with IOP (Fig. 1M, P-value = 0.000961).

Table 2 lists the area under the curve (AUC) values of receiver operating characteristic curves for differentiation between normal and glaucoma groups. We also analyzed whether the TGF-β/ATX ratio could be used to differentiate among glaucoma subtypes. The level of ATX had a high AUC value for differentiating between glaucoma and control groups, as well as between POAG and SOAG groups, and POAG and XFG groups. The level of TGF-β1 was also useful for differentiating between glaucoma and control groups, and between POAG and XFG groups, but the TGF-β1/ATX ratio did not have a higher AUC than TGF-β1 alone. The level of TGF-β2 was useful for differentiating between control and POAG groups, and between XFG and POAG or SOAG groups, but could not be used to differentiate between POAG and SOAG groups. The TGF-β2/ATX ratio could be used to differentiate between control and glaucoma groups (P = 0.0036), POAG and SOAG groups (P = 0.0284), POAG and XFG groups (P = 0.0027), and SOAG and XFG groups (P = 0.039). The TGF-β3/ATX ratio could be used to differentiate between POAG and XFG groups (P = 0.0023), as well as between SOAG and XFG groups (P = 0.001). The level of TGF-β3 could be used to differentiate between XFG and POAG or SOAG groups. Similarly, the TGF-β3/ATX ratio could clearly be used to differentiate between XFG and POAG or SOAG groups.

**Table 2:** AUC values for classifying disease types

Aqueous mediator	Control and glaucoma	Control and POAG	POAG and SOAG	POAG and XFG	SOAG and XFG
ATX	0.8569**	0.72353*	0.79474**	0.82895 **	0.5928
TGFβ1	0.84636**	0.76324	0.68824	0.85833**	0.71732
TGFβ2	0.58409	0.78125*	0.62353	0.84722**	0.73203*
TGFβ3	0.75273	0.43676	0.66029	0.98056 **	0.91503**
TGFβ1/ATX	0.51818	0.54706	0.4671	0.77222**	0.7451
TGFβ2/ATX	0.72543**	0.49118	0.77353*	0.93889 **	0.71895*
TGFβ3/ATX	0.58091	0.61765	0.58529	0.97778**	0.92157**

*P < 0.05, **P < 0.01

*ATX* autotaxin, *TGFβ* transforming growth factor-beta, *POAG* primary open angle glaucoma, *SOAG* secondary open angle glaucoma, *XFG* Exfoliation glaucoma

### Correlations between ATX and TGF-β1–3

Next, we evaluated the correlations of the aqueous level of ATX with aqueous levels of TGF-β1, TGF-β2, and TGF-β3 (Additional file 1: Figure S2). Among TGF-βs, a significant correlation was observed only between the levels of TGF-β1 and TGF-β3 (P < 0.0001). Analysis of the levels of ATX and TGF-βs revealed that the levels of TGF-β1 (P < 0.0001) and TGF-β3 (P < 0.0001) were positively correlated with the level of ATX (Additional file 1: Figure S2A and C), while the level of TGF-β2 was not (Additional file 1: Figure S2B, P = 0.771). We also evaluated the correlations in specific glaucoma subtypes. For the POAG group (Additional file 1: Figure S2D–F), the level of ATX was positively correlated with levels of TGF-β1 (Additional file 1: Figure S2D, P = 0.00494) and TGF-β2 (Additional file 1: Figure S2E, P = 0.0408), but not with the level of TGF-β3 (Additional file 1: Figure S2F). For the XFG group (Additional file 1: Figure S2J–L), levels of TGF-β1 (Additional file 1: Figure S2J, P < 0.0001) and TGF-β3 (Additional file 1: Figure S2L, P = 0.0192) were positively correlated with the level of ATX, while the level of TGF-β2 was not (Additional file 1: Figure S2K, P = 0.761). For the SOAG group (Additional file 1: Figure S2G–I), no significant correlations were observed between the level of ATX and levels of TGF-β1–3.

### TGF-β2 and ATX mRNA expression levels in anterior segments of normal and SOAG eyes

**Fig. 2:**
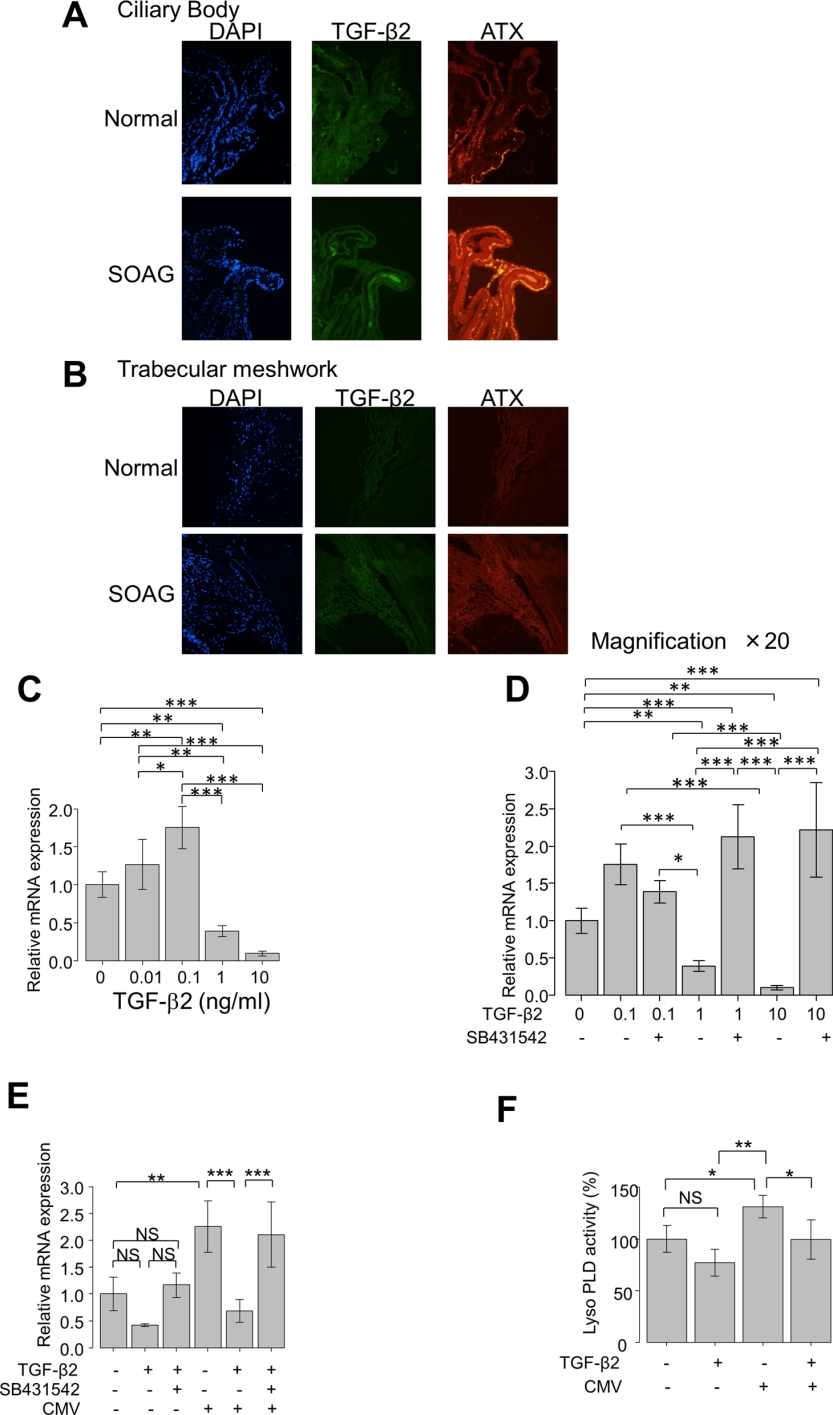
(**A**, **B**): RNAscope analysis of TGF-β2 and ATX mRNA expression levels in anterior segments of normal and SOAG eyes. Expression levels of TGF-β2 and ATX in CB (**A**) or TM (**B**) of normal and SOAG eyes. Both TGF-β2 and ATX mRNA expression levels were detectable in CB (**A**) and TM (**B**) in anterior segments of normal eyes. Expression levels of both TGF-β2 and ATX in CB were enhanced in SOAG eyes compared with normal eyes, while the expression level of ATX was elevated in CB of SOAG eyes (**A**). Expression levels of TGF-β2 and ATX in TM exhibited similar tendencies, and the expression level of ATX was also more robust than that of TGF-β2 in TM of SOAG eyes (**B**). **C**–**F** Effects of TGF-β2 on ATX mRNA expression level in hTM cells and LysoPLD activity in cell culture supernatant (n = 4). The relative mRNA expression level of ATX was significantly upregulated, compared with controls, following treatment with 0.1 ng/ml TGF-β2, but this upregulation was significantly suppressed by treatment with TGF-β2 at a concentration > 1 ng/ml (**C**). These effects were significantly suppressed by treatment with TGF-β inhibitor SB431542 (**D**). **E** Following treatment with TGF-β2 at a concentration of 500 pg/ml, the expression level of ATX was not significantly changed. The expression of ATX was significantly upregulated with CMV infection in the absence of TGF-β2, while the expression of ATX was significantly downregulated in the presence of 500 pg/ml TGF-β2. This downregulation was suppressed by treatment with TGF-β inhibitor (**E**). **F** LysoPLD activity in the conditioned medium was significantly upregulated by CMV infection, and suppressed by treatment with 500 pg/ml TGF-β2. ^*^P < 0.05, ^**^P < 0.01, ^***^P < 0.001

We sought to elucidate the role and crosstalk of TGF-β2 and ATX in glaucoma pathogenesis and regulation of TM fibrosis. First, using RNAscope analysis, we assessed mRNA expression levels and co-localization of TGF-β2 and ATX in donor eyes from normal individuals or patients with SOAG. As shown in Fig. 2, both TGF-β2 and ATX mRNA expression levels were detectable in the ciliary body (CB) and TM in the anterior segments of normal eyes. The expression levels of both TGF-β2 and ATX in CB were enhanced in SOAG eyes, compared with normal eyes. The expression level of ATX was elevated in CB of SOAG eyes (Fig. 2A). The expression levels of TGF-β2 and ATX in TM exhibited similar tendencies, and the expression level of ATX was stronger than that of TGF-β2 in TM of SOAG eyes (Fig. 2B). These findings are consistent with the profiles of these mediators in AH of patients with SOAG.

### Effects of TGF-β2 on mRNA expression of ATX in human TM cells

Next, we investigated the effects of TGF-β2 on mRNA expression of ATX in cultured human TM (hTM) cells in vitro. The mRNA levels in hTM cells were assessed by quantitative polymerase chain reaction (qPCR) (Fig. 2C, D), following treatment with various concentrations of TGF-β2 (0–10 ng/ml). The relative mRNA expression of ATX was significantly upregulated, compared with controls, when hTM cells were treated with 0.1 ng/ml TGF-β2. However, this upregulation was significantly suppressed, compared with controls, when the TGF-β2 concentration was > 1 ng/ml (Fig. 2C). These effects were significantly suppressed by treatment with TGF-β inhibitor SB431542 (Fig. 2D). Thus, ATX upregulation in hTM cells was regulated by TGF-β2 in a concentration-dependent manner.

We previously reported that CMV infection could upregulate ATX expression in hTM cells, thus mimicking SOAG pathology in vitro [18]. Therefore, we explored whether the upregulation of ATX in CMV-infected hTM cells could be affected by pre-treatment with TGF-β2. hTM cells were pre-stimulated with 500 pg/ml TGF-β2, a modified aqueous TGF-β2 concentration based on the clinically active TGF-β2 levels in AH. As shown in Fig. 2E, at 500 pg/ml TGF-β2, the expression of ATX was neither upregulated nor suppressed significantly. The ATX expression was significantly upregulated with CMV infection in the absence of TGF-β2, but the expression of ATX was significantly downregulated in the presence of TGF-β2. This downregulation was suppressed by treatment with TGF-β inhibitor (Fig. 2E).

To confirm the activity of ATX secreted from hTM cells, we measured lysophospholipase D (lysoPLD) activity (the ability of ATX to generate LPA) in the conditioned medium (Fig. 2F). Consistent with the regulation of ATX mRNA expression shown in Fig. 2E, the lysoPLD activity in the conditioned medium was significantly upregulated by CMV infection, and suppressed in the presence of TGF-β2.

### Immunocytochemical analysis of the effects of TGF-β2 on protein expression of ATX in hTM cells

**Fig. 3:**
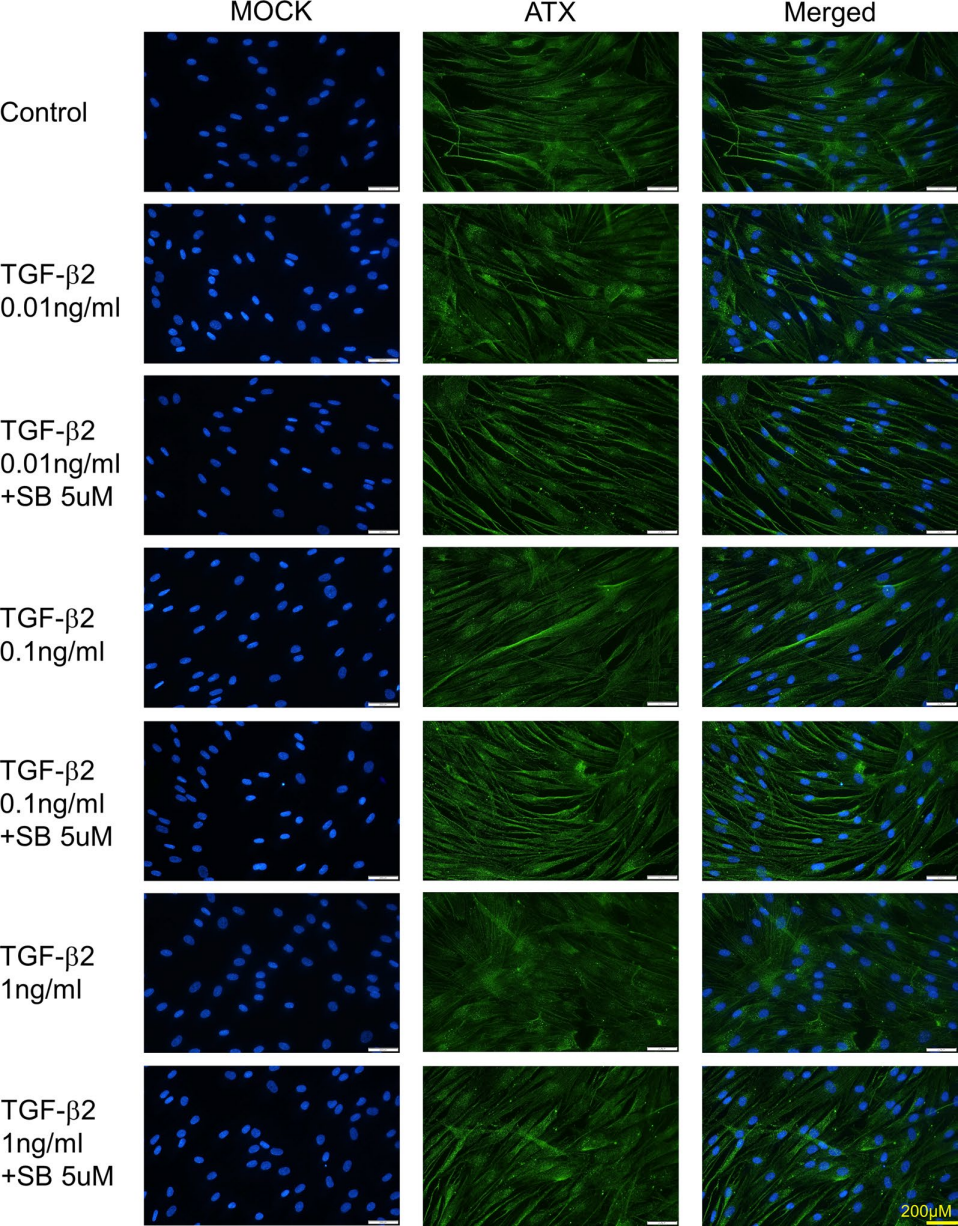
Confirmation of the effects of TGF-β2 on ATX protein expression in hTM cells by immunocytochemistry. Immunocytochemistry of ATX in TGF-β2-stimulated hTM cells. Left panels present cells that were stained with DAPI (4’,6-diamidino-2-phenylindole). Middle panels present cells stained for ATX. Right panels present merged images. Characteristic ATX protein expression in vesicles was evident after treatment with TGF-β2 at concentrations of 0.01 and 0.1 ng/ml, while ATX protein expression was downregulated after treatment with TGF-β2 at the concentration of 1 ng/ml. These effects were significantly suppressed by treatment with the TGF-β inhibitor SB431542. Bar, 200 µm

Immunocytochemistry was used to confirm the concentration-dependent effects of TGF-β2 on ATX protein expression patterns in hTM cells. As shown in Fig. 3, the characteristic protein expression pattern of ATX in vesicles was considerably enhanced after TGF-β2 treatment at concentrations of 0.01 and 0.1 ng/ml, but ATX protein expression was reduced after TGF-β2 treatment at the concentration of 1 ng/ml. The concentration-dependent changes in ATX expression induced by TGF-β2 were significantly suppressed by treatment with TGF-β inhibitor.

### Effects of TGF-β2 on ATX-related transcription factors in hTM cells

**Fig. 4:**
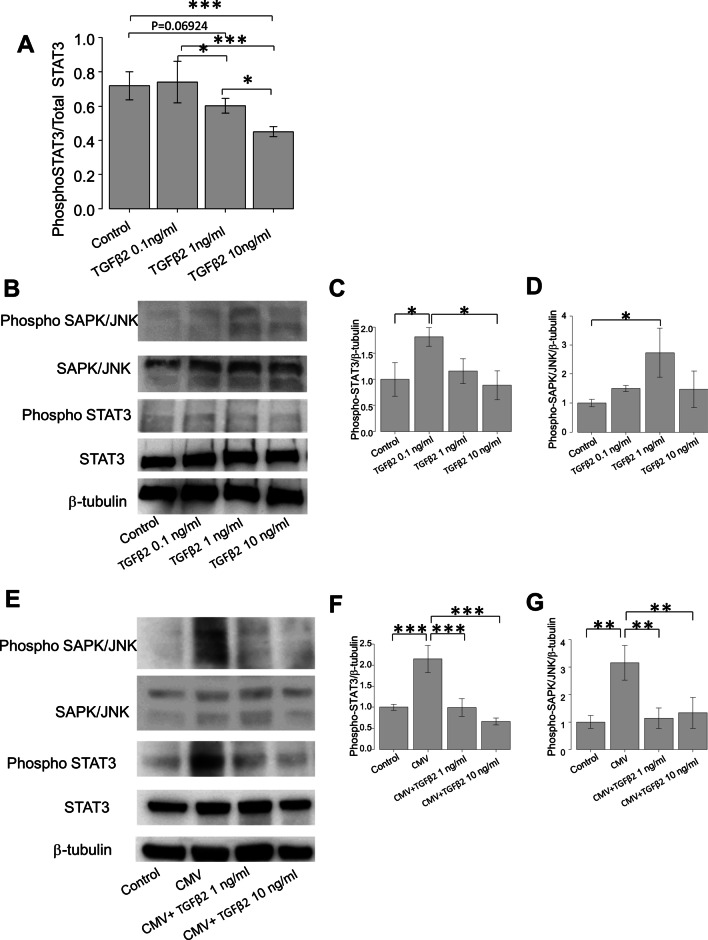
Effects of TGF-β2 on phosphorylation of STAT3 or SAPK/JNK in hTM cells. **A** Enzyme-linked immunosorbent analysis revealed significant downregulation of phospho-STAT3/total STAT3 ratio in hTM cells after treatment with TGF-β2 at the concentration of 10 ng/ml, compared with control or concentrations of 0.1 and 1 ng/ml. The ratio was also significantly downregulated after treatment with TGF-β2 at the concentration of 1 ng/ml, compared with 0.1 ng/ml (n = 4). **B**–**D** Western blotting of stress-activated protein kinase (SAPK)/Jun amino terminal kinase (JNK) and STAT3 in TGF-β2 treated hTM cells (n = 3). Representative bands for SAPK/JNK and STAT3 are shown in (B), while the relative expression levels of STAT3 (C) and SAPK/JNK (D) are shown compared with the loading control (β-tubulin) (n = 3). Phospho-STAT3 was significantly upregulated when hTM cells were treated with 0.1 ng/ml TGF-β2 compared with control, and downregulated compared with 10 ng/ml TGF-β2 (C). Phospho-SAPK/JNK was significantly upregulated when hTM cells were treated with 1 ng/ml TGF-β2. Quantitative analysis also revealed significant differences (**D**). ^*^P < 0.05, ^**^P < 0.01, ^***^P < 0.01. (E–G) Western blotting of stress-activated protein kinase (SAPK)/Jun amino terminal kinase (JNK) and STAT3 in TGF-β2 treated CMV-infected hTM cells (n = 3). Representative bands for SAPK/JNK and STAT3 are shown in **E**, while the relative expression levels of STAT3 (**F**) and SAPK/JNK (**G**) are shown compared with the loading control (β-tubulin) (n = 3). Phospho-STAT3 was significantly upregulated when hTM cells were infected with CMV compared with control, and this change was attenuated by treatment with 1 ng or 10 ng/ml TGF-β2 (**F**). Phospho-SAPK/JNK was significantly upregulated when hTM cells were infected with CMV compared with control, and this change was attenuated by treatment with 1 ng or 10 ng/ml TGF-β2 (**F**). Quantitative analysis also revealed significant differences. ^*^P < 0.05, ^**^P < 0.01, ^***^P < 0.001

To investigate the effects of TGF-β2 on the transcriptional regulation of ATX in hTM cells, we investigated the phosphorylation statuses of signal transducer and activator of transcription 3 (STAT3) and stress-activated protein kinase (SAPK)/Jun amino-terminal kinase (JNK), as these transcription factors reportedly regulate ATX expression [24, 25]. Using enzyme-linked immunosorbent analysis, we observed significant downregulation of the phospho-STAT3/total STAT3 ratio in hTM cells after TGF-β2 treatment at the concentration of 10 ng/ml, compared with control or 0.1 and 1 ng/ml concentrations of TGF-β2. This ratio was also significantly downregulated after TGF-β2 treatment at the concentration of 1 ng/ml, compared with 0.1 ng/ml (Fig. 4A). Western blotting (WB) analysis revealed that phospho-STAT3 was significantly upregulated after treatment with 0.1 ng/ml TGF-β2, but no significant differences were observed between control and 1 or 10 ng/ml concentrations of TGF-β2 (Fig. 4B, C). Significant upregulation of phospho-SAPK/JNK was observed when hTM cells were treated with 1 ng/ml TGF-β2 (Fig. 4D).

### Effects of TGF-β2 on phosphorylation of STAT3 and SAPK/JNK in CMV-infected hTM cells

Figure 4E presents the expression levels of STAT3 and SAPK/JNK when hTM cells were infected with CMV, in the presence or absence of TGF-β2. Expression levels of both phospho-STAT3 and phospho-SAPK/JNK were significantly upregulated with CMV infection, whereas they were significantly attenuated in the presence of TGF-β2 at the concentrations of 1 and 10 ng/ml (Fig. 4F and 4G, P < 0.001 for phospho-STAT3 and P < 0.01 for phospho-SAPK/JNK).

### Effects of canonical TGF-β pathway on the expression of ATX in hTM cells

**Fig. 5:**
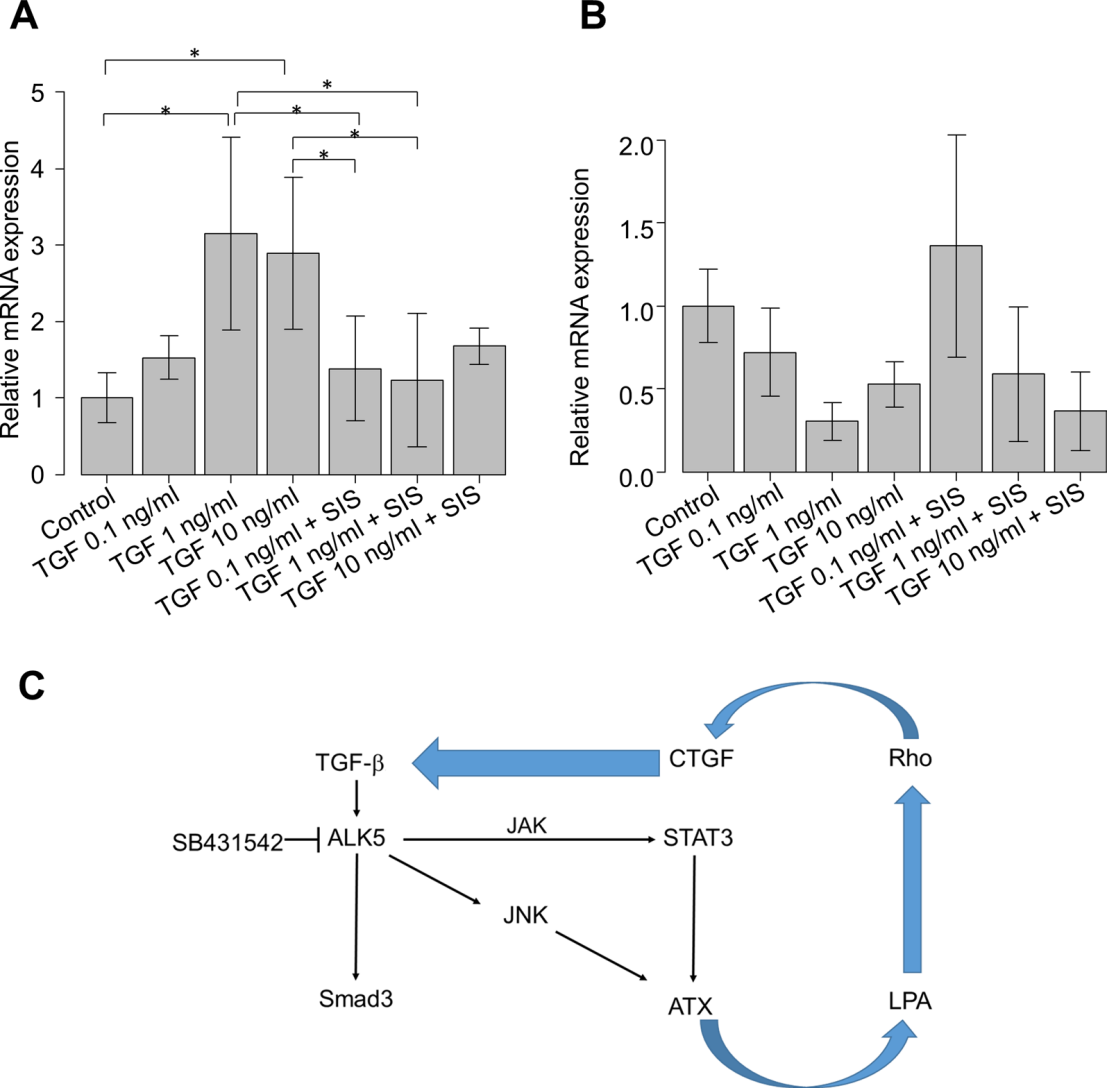
Effects of Smad3 inhibitor (SIS3) under TGF-β2 stimulation on the mRNA expression levels of CTGF and ATX (n = 4). After treatment with various concentrations of TGF-β2 (0–10 ng/ml), the relative mRNA expression level of CTGF was significantly upregulated compared with controls, but this upregulation was significantly suppressed by treatment with 10 μM SIS3 (**A**). In contrast, the expression level of ATX did not change after treatment with SIS3 (**B**). ^*^P < 0.05, ^**^P < 0.01, ^***^P < 0.001. **C** Possible mechanisms of TGF-β2-medated ATX expression in hTM cells. Expression of ATX may be mediated through a non-canonical TGF-β pathway, but not the canonical TGF-β pathway

Because the non-canonical TGF-β pathway is presumably involved in ATX expression, we investigated the effects of TGF-β2 on ATX in the presence or absence of 10 μM Smad3 inhibitor (SIS3). As shown in Fig. 5A, the expression of connective tissue growth factor (CTGF) was significantly upregulated with TGF-β2 stimulation in a concentration-dependent manner, whereas it was significantly downregulated with 10 μM SIS3 application. In contrast, the expression of ATX was not affected by SIS3 application (Fig. 5B).

### Effects of ATX on expression levels of TGF-βs

**Fig. 6:**
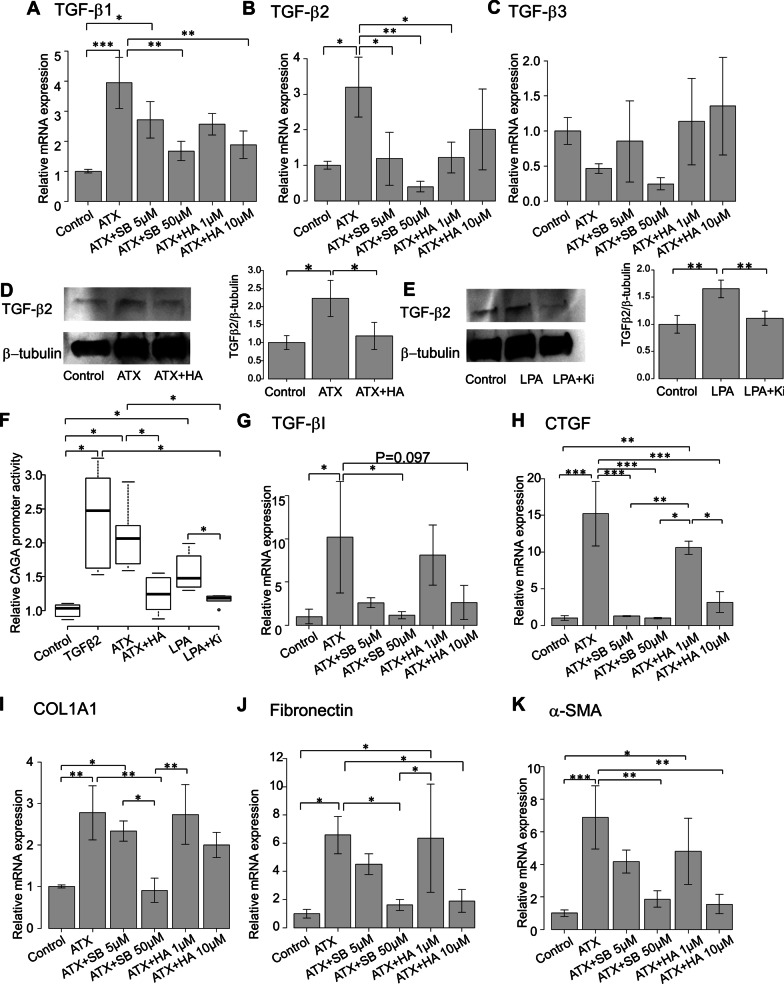
Effects of ATX on the expression levels of TGF-βs. **A**–**C** n = 4) Relative mRNA expression levels of TGF-β1–3 in hTM cells were evaluated following ATX stimulation. The expression of TGF-β1 was significantly upregulated with ATX stimulation, and this change was significantly attenuated by treatment with 10 μM SB431542 or 10 μM HA130 (**A**). The expression of TGF-β2 was also significantly upregulated with ATX stimulation, and this change was attenuated by treatment with 5 or 50 μM SB431542, or 1 μM HA130 (**B**). However, the expression of TGF-β3 did not significantly differ following ATX stimulation (C). ^*^P < 0.05, ^**^P < 0.01, ^***^P < 0.001. **D**–**E** Western blotting analysis of TGF-β2 when hTM cells were stimulated with ATX (D) or LPA (**E**). Figures on the right side present representative WB bands when hTM cells were stimulated with 40 μM ATX or 10 μM LPA with or without corresponding inhibitors (10 μM HA130 or 10 μM Ki16425). The expression of TGF-β2 was upregulated upon stimulation with ATX or LPA, and this change was attenuated by treatment with the corresponding inhibitors (**D** and **E**). Quantitative analysis revealed significant differences (n = 3, **E** and **G**). ^*^P < 0.05, ^**^P < 0.01. ATX induced expression of CAGA-12 promoter activity, TGF-β-induced protein and CTGF and fibrotic markers in hTM cells (F-K, n = 4). **F** Luminescence in hTM cells expressing a luciferase reporter gene fused with 12 repeats of the Smad-binding element (CAGA-12) was measured using luciferase assay. Relative activity of the promoter was measured under treatment with 10 ng/ml TGF-β2, 40 µM ATX with or without its inhibitor (10 µM HA130), and 10 µM LPA with or without its inhibitor (10 µM Ki16425). The relative levels of CAGA-12 promoter activity were significantly upregulated after treatment with TGF-β2, ATX, and LPA, and those changes were significantly downregulated when either ATX inhibitor (HA130) or LPA inhibitor (Ki16425) were applied (**F**). ^*^P < 0.05. **G** The relative mRNA expression level of TGF-β-induced protein was evaluated following ATX stimulation in hTM cells. The expression of TGF-β-induced protein was significantly upregulated with ATX stimulation, and this change was inhibited by treatment with 50 μM SB431542 but not ATX inhibitor (**G**). ^*^P < 0.05. (H) Measurement of ATX-induced CTGF mRNA expression. CTGF was significantly upregulated by ATX treatment, and the changes were attenuated by inhibition of TGF-β or ATX. ^*^P < 0.05, ^**^P < 0.01, ^***^P < 0.001. (I-K): Effects of ATX on the mRNA expression levels of fibrotic markers. Relative mRNA expression levels of COL1A1, fibronectin, and α-SMA were analyzed under ATX stimulation with or without 5 μM or 50 μM SB431542, or 1 μM or 10 μM HA130. Expression levels of COL1A1, fibronectin, and α-SMA were enhanced upon ATX stimulation, and these changes were significantly suppressed by treatment with either TGF-β inhibitor or ATX inhibitor (**D**, **E**, and **F**)

Next, we investigated whether ATX affects the expression levels of TGF-βs. The relative mRNA expression levels of TGF-β1–3 were evaluated in hTM cells subjected to ATX stimulation. The expression of TGF-β1 was significantly upregulated with ATX stimulation, while this change was significantly attenuated by treatment with 10 μM SB431542 or 10 μM HA130 (P < 0.01; Fig. 6A). The expression of TGF-β2 was also significantly upregulated with ATX stimulation, but this change was attenuated by treatment with 5 or 50 μM SB431542, or 1 μM HA130 (P < 0.05 for 5 μM SB431542 and 1 μM HA130, P < 0.01 for 50 μM SB431542; Fig. 6B). However, the expression of TGF-β3 did not differ significantly following ATX stimulation (Fig. 6C). Subsequently, we used WB to confirm that activation of the ATX signaling pathway influenced TGF-β2 expression. Figure 6D and E present the representative bands for WB when hTM cells were stimulated with 40 μM ATX or 10 μM LPA, in the presence or absence of the corresponding inhibitors (10 μM HA130 or 10 μM Ki16425). The expression of TGF-β2 was upregulated following stimulation with ATX or LPA, and this change was attenuated by treatment with the corresponding inhibitors (Fig. 6D and E). Quantitative analysis also revealed significant differences (n = 3, Fig. 6D and E). Collectively, these results indicate that TGF-β1 and 2 were upregulated by ATX stimulation, and that this upregulation was suppressed by both TGF-β inhibition and ATX inhibition. Thus, ATX might transactivate TGF-β in hTM cells.

### Effects of ATX/LPA pathway on transactivation of TGF-β

To clarify the effects of ATX transactivation on the transcriptional activity of the Smad regulator, we measured the luminescence from a luciferase reporter gene fused with 12 repeats of the Smad-binding element (CAGA-12) using luciferase assay in hTM cells, as previously described [20, 21, 26]. We measured the relative CAGA-12 promoter activity under treatment with 10 ng/ml TGF-β2, 40 µM ATX with or without its inhibitor (10 µM HA130), and 10 µM LPA with or without its inhibitor (10 µM Ki16425). The relative levels of CAGA-12 promoter activity were significantly upregulated after treatment with TGF-β2, ATX, and LPA. Those changes were significantly downregulated when either ATX inhibitor (HA130) or LPA inhibitor (Ki16425) were applied (Fig. 6F).

Because treatment with ATX significantly upregulated expression levels of TGF-β1, we explored the expression of TGF-β-induced protein, as TGF-β-induced protein inhibition reportedly increased TGF-β1 release and led to TGF-β1 pathway activation [27]. However, the expression of TGF-β-induced protein was significantly upregulated with ATX stimulation (P < 0.05; Fig. 6G), and the change was inhibited by treatment with 50 μM SB431542 (P < 0.05; Fig. 6G) but not by ATX inhibitor. These findings indicate that the regulation of TGF-β1 was mediated by mechanisms other than integrin-mediated regulation involving TGF-β-induced protein.

LPA reportedly transactivates latent TGF-β in a Rho-CTGF-dependent manner [28], and Smad-mediated CTGF activation is presumed to be involved in positive forward feedback of TGF-β [29]. Therefore, we investigated ATX-induced mRNA expression of CTGF. As shown in Fig. 6H, CTGF was significantly upregulated by ATX treatment, while the changes were attenuated via inhibition of TGF-β or ATX, implying that ATX/LPA-induced transactivation of TGF-β could be induced by downstream CTGF signaling. These results indicate that the activated ATX/LPA pathway transactivated the TGF-β/Smad/CTGF signaling pathway and regulated positive feedback of TGF-β in hTM cells.

### Effects of ATX and ATX trans-signaling on mRNA expression levels of fibrotic markers

To elucidate the effects of ATX trans-signaling on fibrogenic changes in hTM cells, we investigated the expression levels of COL1A1, fibronectin, and α-SMA (representative markers of fibrosis and epithelial-to-mesenchymal transition). The relative mRNA expression levels of COL1A1, fibronectin, and α-SMA under ATX stimulation in the presence or absence of 5 or 50 μM SB431542, or 1 or 10 μM HA130, were analyzed using reverse transcriptase (RT)-qPCR.

ATX-induced changes in COL1A1, fibronectin, and α-SMA were significantly suppressed by treatment with either TGF-β inhibitor or ATX inhibitor (Fig. 6I, J, and K). These results indicate that ATX-induced fibrosis and epithelial-to-mesenchymal transition were at least partly mediated by transactivated TGF-β signaling in hTM cells.

## Discussion

In this study, we investigated the aqueous concentrations of ATX and TGF-βs among glaucoma subtypes and found significant differences in the profiles of these mediators among groups, as well as significant correlations among specific mediators in each group. TGF-β2 has been presumed to play a critical role in POAG pathogenesis based on its elevated levels in AH and its abilities to induce ECM remodeling and TM fibrosis [30], but the molecular mechanisms involved in generating a glaucomatous environment remain unknown. Investigation of the molecular mechanism responsible for crosstalk between TGF-β2 and ATX in hTM cells further elucidated the possible mechanisms involved in the glaucomatous profile of AH and pathogenesis in TM.

Our clinical study of AH in patients with glaucoma demonstrated that the level of ATX was significantly higher in SOAG or XFG groups than in control or POAG groups, whereas the level of TGF-β2 was downregulated in AH in an inverse manner, consistent with the results of previous reports [4, 10–12]. However, the level of TGF-β2 was not correlated with other mediators or IOP in the overall cohort. The level of TGF-β2 was correlated with the level of ATX only when limited to the POAG group (Figure S1E), supporting the specific role of TGF-β2 in POAG pathogenesis.

AH levels of TGF-β1and TGF-β3 exhibited similar tendencies to ATX levels. These three mediators were correlated with each other, and were positively correlated with IOP in the overall cohort. Furthermore, although TGF-β1 has been implicated in glaucoma through genetic analysis, and has been presumed to play a role in pathological IOP elevation through fibrotic regulation of TM and ECM remodeling [31, 32], it has been speculated that the high IOP itself might induce the expression of activated TGF-β1 in TM cells. Thus far, the pathological role of TGF-β1 in glaucoma remains unclear [33]. We and others have reported that CMV infection induces significant TGF-β1 upregulation in hTM cells and could be related to the enhanced fibrotic response in TM [18, 34, 35]. However, we recently confirmed that ATX expression was significantly induced by CMV infection preceding the upregulation of TGF-β1 expression in TM. Moreover, the level of TGF-β1 was not correlated with IOP, while the level of ATX was significantly positively correlated with IOP [18]. In the present study, we found that treatment with ATX significantly upregulated TGF-β1 expression in hTM cells (Fig. 6A). RNAscope analysis revealed extensive mRNA localization of ATX in CB (Fig. 2A), where AH is generated. Therefore, we speculate that AH with a high concentration of ATX could be generated in SOAG eyes, which then affects TM and contributes to the high expression of TGF-β1 in SOAG.

Higher levels of TGF-β3 have been observed in a few studies regarding XFG, but to date little is known about the role of TGF-β3 in glaucoma pathogenesis [36, 37]. XFG is one of the glaucoma subtypes which is usually categorized in SOAG. However, at the same time, XFG causes more severe inflammation and excessive fibrosis in TM with elevated IOP [10, 38, 39], and its prognosis is poor compared to other subtypes which are categorized as SOAG. In our previous reports, we have reported that there were significant differences concerning the upregulation of aqueous ATX between SOAG and XFG, and furthermore, most recently, we have reported the diagnostic ability of these characteristic upregulation of mediators in AH that high AUC values were obtained with ATX for discriminating XFG from normal eyes [12, 40]. Therefore, we focused and enrolled XFG patients apart from SOAG in this study as an independent group. Although the level of TGF-β3 was correlated with the levels of TGF-β1 and ATX, we found no correlations between TGF-β3 and TGF-β2, and ATX treatment did not affect the expression of TGF-β3 (Additional file 1: Figure S2, Fig. 6C). TGF-β3 might have a pathological role in XFG, but presumably does not have a role in POAG or SOAG. Collectively, the results indicate that TGF-β1 or TGF-β3 could be at least partly involved in glaucomatous changes in the TM, but they are unlikely to play a predominant role in the glaucomatous eye. We previously observed that significantly reduced expression of TGF-β2 was induced by CMV infection [18], which implied that TGF-β2 and ATX are important factors in SOAG (e.g., via crosstalk between ATX and TGF-β2).

We also analyzed the diagnostic value of levels of aqueous ATX and TGF-βs by comparing the AUC values (Table 2). Consistent with previous findings, the level of ATX was of high diagnostic value in differentiating the POAG group from the SOAG or XFG groups [12]. The level of TGF-β2 was also of high diagnostic value in differentiating the POAG group from the XFG group. Analysis of TGF-β2 and ATX revealed that the TGFβ2/ATX ratio could effectively be used to differentiate the POAG group from the SOAG or XFG groups (Fig. 1J and Table 2). In addition to the investigation on the correlation between aqueous mediators and IOP or glaucoma subtypes, the relationship between the mediators and glaucoma progression is another important issue. Most recently, we reported the relationship between the aqueous mediators (ATX and TGF-b1-3) and mean deviation (MD), using aqueous humor samples obtained from 281 consecutive patients [40]. We found that ATX was the only factor that exhibited a negative correlation to the MD, and TGF-β1–β3 did not exhibit any significant correlations. It is possible to speculate that ATX reflect IOP elevation and could be an aqueous biomarker which may predict the possible progression of glaucoma reflecting the IOP elevation, however, not only the data from visual field testing (MD) but also results from optical coherence tomography (OCT) would be needed to further understand the relationship between aqueous mediators and glaucoma progression. We would like to investigate this issue in the future study.

Accordingly, we speculated that more crosstalk might be present between TGF-β2 and ATX, and we next investigated this using in vitro analysis.

We first performed RNAscope analysis to identify the mRNA localization of TGF-β2 and ATX in the anterior segment of the eye (Fig. 2A and B). We confirmed increased expression levels of both mediators in CB and TM in normal and SOAG eyes. The expression levels of both TGF-β2 and ATX were upregulated in CB and TM in SOAG eyes, compared with normal eyes. Moreover, ATX expression was further enhanced, compared with TGF-β2. As mentioned above, the increased expression of ATX in CB of SOAG eyes resulted in the production of AH with a high ATX concentration in patients with SOAG. The mRNA expression level of ATX was also high in TM of SOAG eyes, while the expression of TGF-β2 was limited. We previously reported that conditions mimicking SOAG, such as dexamethasone stimulation or CMV infection [12, 18], induced ATX protein expression and activity in hTM cells. Additionally, those hTM cells secreted ATX and exhibited upregulation of lysoPLD activity in the medium, affecting TM fibrosis in an autocrine or paracrine manner. Given these results, the AH generated from ATX-enhanced CB and ATX secreted from challenged hTM cells presumably synergistically contribute to a high concentration of ATX in AH, leading to glaucomatous changes in the aqueous pathway in SOAG eyes.

Notably, the mRNA and protein expression levels of ATX in hTM cells were significantly upregulated by exogenous TGF-β2 treatment at the lower concentration of < 0.01 ng/mL, but significantly downregulated at the concentration of 1 ng/mL (Fig. 2C). An inverse relationship was also observed. These findings highlight the specific differences in AH concentrations of TGF-β2 among glaucoma subtypes. We investigated whether the CMV-infection-induced upregulation of ATX was affected by the presence of TGF-β2, and found that the upregulated mRNA expression and lysoPLD activity of ATX were significantly attenuated in the presence of TGF-β2 (Fig. 2E and F). We used 500 pg/mL TGF-β2 to modify the effective TGF-β2 levels in AH, such that the mean values of aqueous total TGF-β2 were 1751.3 ± 452.6 pg/mL in the control group and 2293.3 ± 592.7 pg/mL in the POAG group (Fig. 1C). We suspect that ATX upregulation evoked by pathological challenge may lead to SOAG, and that this is suppressed in environments with higher levels of TGF-β2. These conditions were present in control and POAG groups in the present study. Importantly, when the level of TGF-β2 is not fully upregulated and the expression of ATX is not fully downregulated (i.e., SOAG or XFG), ATX is not expected to be suppressed. This induces excessive ECM deposition and TM fibrosis, leading to an overwhelming increase in IOP.

To clarify the transcriptional regulatory mechanisms of TGF-β2 with regard to ATX expression, we analyzed the effects of TGF-β2 on two reported modulators of ATX, SAPK/JNK and STAT3, which are involved in non-canonical TGF-β signaling. In the culture medium, the ratio of phospho-STAT3 relative to total STAT3 was significantly downregulated upon treatment with 1 ng/mL TGF-β2 (Fig. 4A). Although we observed no significant difference compared with control at the TGF-β2 concentration of < 0.1 ng/mL, the expression of phospho-STAT3 tended to be upregulated. This concentration-dependent effect of TGF-β2 on phospho-STAT3 expression coincided with the changes in mRNA expression level of ATX induced by treatment with TGF-β2 (Fig. 2C). It has been suggested that STAT3 is a direct transcriptional regulator of ATX and that downregulation of STAT3 causes a significant reduction in ATX protein expression in human tumor cells [24, 25]. Our data suggest that ATX upregulation and downregulation in hTM cells is likely mediated by STAT3 activation. This was confirmed by our WB analysis (Fig. 4B–D), whereby the expression of phospho-STAT3 was significantly upregulated upon treatment with TGF-β2 at the concentration of 0.1 ng/mL, while it was downregulated upon treatment with TGF-β2 at the concentration of < 0.1 ng/mL. The expression of phospho-SAPK/JNK, which is reportedly activated by non-canonical TGF-β signaling and crosstalk with STAT3 [41], was also upregulated upon treatment with TGF-β2 at the concentration of 1 ng/mL. Treatment with a higher concentration of TGF-β2 tended to downregulate the expression of phospho-SAPK/JNK (Fig. 4B and D).

We also examined how these transcriptional factors are mediated by TGF-β2 in the pathologic condition where the expression of ATX is exogenously upregulated. During CMV infection of hTM cells, SAPK/JNK-STAT3 signaling was significantly activated, which may lead to ATX upregulation. This activation was significantly attenuated upon treatment with TGF-β2 at the concentration of < 1 ng/mL (Fig. 4E–G). Because we observed clear differences between JNK and STAT3 in the concentration of TGF-β2 required to regulate their activation (Fig. 4C and D), they could be regulated separately in hTM cells, although crosstalk may exist between JNK and STAT3. We also investigated whether the canonical TGF-β pathway is involved in ATX expression via treatment with Smad3 inhibitor (SIS3) under TGF-β2 stimulation. However, we found no effects of the canonical pathway on ATX expression (Fig. 5B).

Collectively, our findings suggest that both JNK and JAK/STAT3 non-canonical pathways are involved in the concentration-dependent TGF-β2 modulation of ATX expression, while the canonical pathways are not involved in ATX expression. A hypothetical model of TGF‐β2‐induced ATX regulation is depicted in Fig. 5C.

We also found that TGF-β1 and 2 were upregulated by exogenous stimulation via the ATX/LPA pathway, and that mechanisms of ATX transactivation involving TGF-β2 were mediated by the TGF-β/Smad/CTGF signaling pathway. It is reported that auto upregulation of TGF-β is mediated by the pathway and forms a positive-feedback circuit [29, 42], and CTGF itself plays an important role in glaucoma pathogenesis by contributing to abnormal accumulation of ECM in TM [43]. Nevertheless, LPA reportedly initiates autocrine signaling and CTGF upregulation, and plays a role in giving rise to paracrine profibrotic signaling in Rho/CTGF dependent activation of TGF-β [28]. We additionally explored the effects of ATX trans-signaling on fibrogenic changes in hTM cells with RT-qPCR (Fig. 6I–K), and expression of COL1A1, fibronectin, and α-SMA by ATX was significantly suppressed by treatment with both TGF-β inhibitor and ATX inhibitor, as expected. These findings suggest that the ATX/LPA pathway-induced fibrosis and epithelial-to-mesenchymal transition could be mediated by transactivated TGF-β/CTGF signaling, at least partly, in pathological conditions.

Although multiple physiological roles have been proposed for TGF-β2 in glaucoma, its clinical significance or signaling pathway regulation remains unclear. Due to the complicated synergistic relationship among the mediators involved in glaucoma pathogenesis, a full understanding of changes in the diseased environment will require further detailed delineation of signaling by multiple classes of G protein-coupled receptors and G proteins.

## Conclusions

The present clinical study is the first to demonstrate possible crosstalk between TGF-β and ATX in the glaucomatous AH, and to investigate the regulatory roles in glaucoma pathogenesis. The results presented here will help to elucidate the molecular mechanisms involved in this extremely complex disease and could provide new targets to lower IOP.

## Supplementary Information

**Additional file 1: Figure S1.** Correlations of IOP with levels of aqueous ATX, TGF-β1, TGF-β2, and TGF-β3 among glaucoma subtypes. **Figure S2.** Correlations between aqueous ATX and TGF-β1–3 in different glaucoma subtypes.

## Data Availability

All data generated or analyzed during this study are included in this published article and its supplementary information files. Reagents used in this publication will be provided upon request.

## Abbreviations

AH: Aqueous humor
AUC: Area under the curve
ATX: Autotaxin
CB: Ciliary body
CTGF: Connective tissue growth factor
CMV: Cytomegalovirus
XFG: Exfoliative glaucoma
ECM: Extracellular matrix
hTM: Human TM
IOP: Intraocular pressure
JNK: Jun amino-terminal kinase
LPA: Lysophosphatidic acid
lysoPLD: Lysophospholipase D
POAG: Primary open-angle glaucoma
qPCR: Quantitative polymerase chain reaction
SOAG: Secondary open-angle glaucoma
STAT3: Signal transducer and activator of transcription 3
SMA: Smooth muscle actin
SAPK: Stress-activated protein kinase
TGF- β: Transforming growth factor-β
TM: Trabecular meshwork
WB: Western blotting
