# Supplementary material for: Crosstalk between transforming growth factor β-2 and Autotaxin in trabecular meshwork and different subtypes of glaucoma
Source: J Biomed Sci. 2021 Jun 17;28:47. doi: 10.1186/s12929-021-00745-3 (PMC8212476; doi:10.1186/s12929-021-00745-3)
Supplement: Supplementary file 1 — Additional file 1: Figure S1. Correlations of IOP with levels of aqueous ATX, TGF-β1, TGF-β2, and TGF-β3 among glaucoma subtypes. Figure S2. Correlations between aqueous ATX and TGF-β1–3 in different glaucoma subtypes. [file 12929_2021_745_MOESM1_ESM.pdf]

Supplemental Figure 1: Correlations of IOP with levels of aqueous ATX, TGF-β1, TGF-β2, and TGF-β3 among glaucoma subtypes.

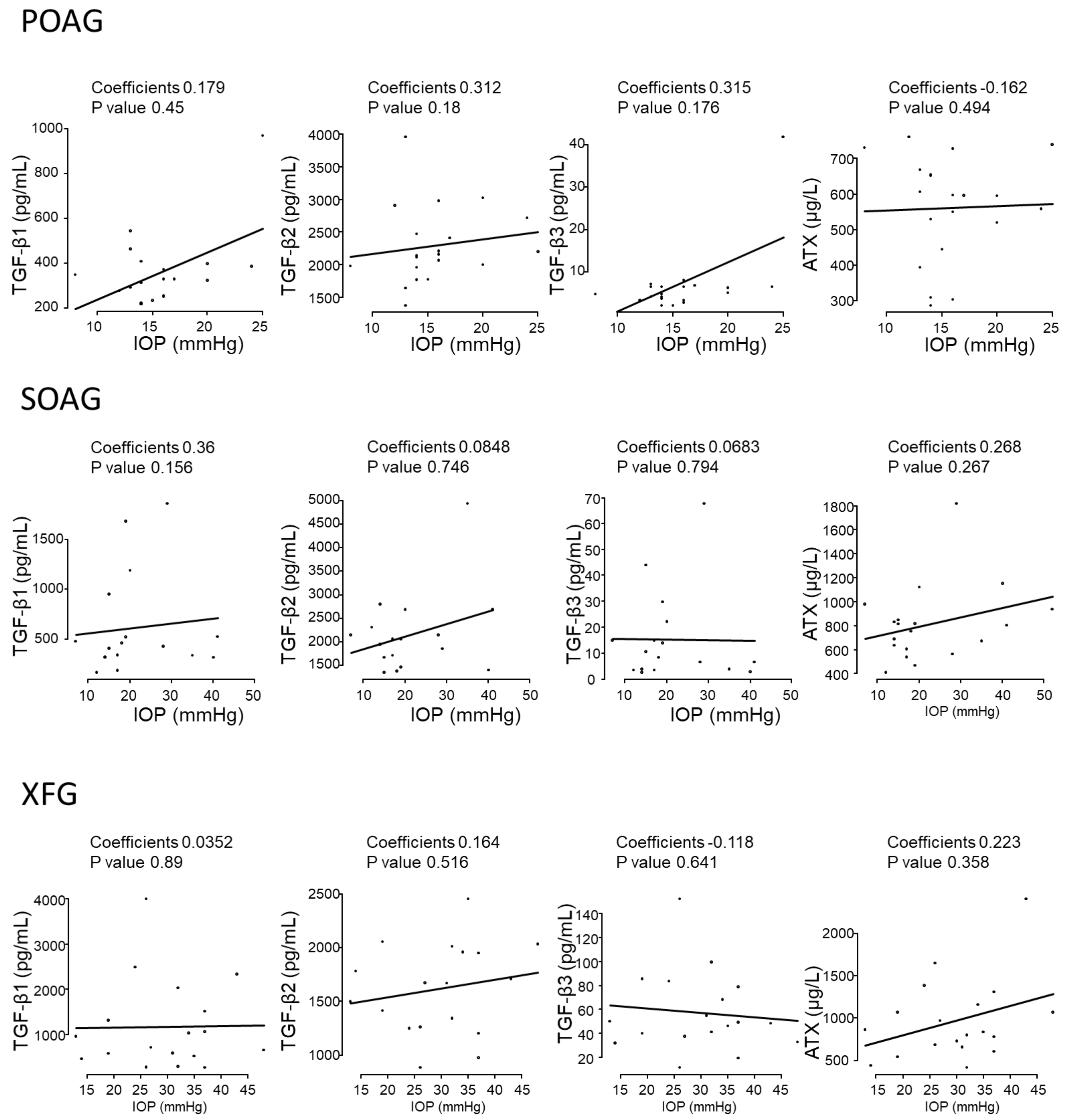

No significant correlations were observed between IOP and levels of aqueous ATX, TGF-β1, TGF-β2, or TGF-β3 when eyes were stratified among glaucoma subtypes.

Supplemental Figure 2: Correlations between aqueous ATX and TGF-β1–3 in different glaucoma subtypes.

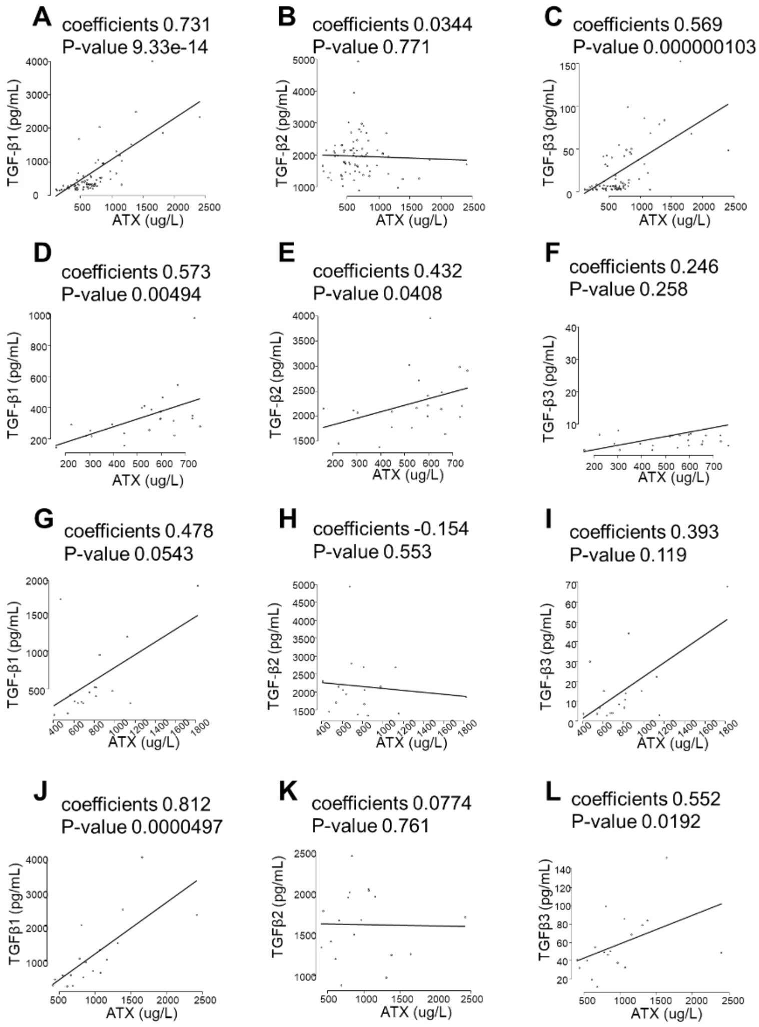

(A–C) Correlations between levels of aqueous ATX and levels of aqueous TGF-β1–3 in the overall cohort. Levels of TGF-β1 (A; Spearman's rank correlation coefficient = 0.731, P = 9.33e-14) and TGF-β3 (B; Spearman's rank correlation coefficient = 0.569, P = 0.000000103) were positively correlated with the level of ATX, whereas the level of TGF-β2 was not (C; Spearman's rank correlation coefficient = 0.0344, P = 0.771).

(D–F) Correlations between levels of aqueous ATX and levels of aqueous TGF-β1–3 in the POAG group. Levels of TGF-β1 (D; Spearman's rank correlation coefficient = 0.573, P = 0.00494) and TGF-β2 (E; Spearman's rank correlation coefficient = 0.432, P = 0.0408) were positively correlated with the level of ATX were positively correlated with ATX, whereas the level of TGF-β3 was not (F; Spearman's rank correlation coefficient = 0.246, P = 0.258).

(G–I) Correlations between levels of aqueous ATX and levels of aqueous TGF-β1–3 in the SOAG group. Levels of TGF-β1 (G; Spearman's rank correlation coefficient = 0.478, P = 0.0543), TGF-β2 (H; Spearman's rank correlation coefficient = -0.154, P = 0.553), and TGF-β3 (I; Spearman's rank correlation coefficient = 0.393, P = 0.119) were not correlated with the level of ATX.

(J–L) Correlations between levels of aqueous ATX and levels of aqueous TGF-β1–3 in the XFG group. Levels of TGF-β1 (J; Spearman's rank correlation coefficient = 0.812, P = 0.0000497) and TGF-β3 (K; Spearman's rank correlation coefficient = 0.552, P = 0.0192) were positively correlated with the level of ATX, whereas the level of TGF-β2 was not (L; Spearman's rank correlation coefficient = 0.0774, P = 0.761).
